# In Vitro Antibacterial and Wound Healing Activities Evoked by Silver Nanoparticles Synthesized through Probiotic Bacteria

**DOI:** 10.3390/antibiotics12010141

**Published:** 2023-01-10

**Authors:** Gayathri Vijayakumar, Hyung Joo Kim, Senthil Kumaran Rangarajulu

**Affiliations:** 1 Department of Biotechnology, Rajalakshmi Engineering College, Chennai 602105, India; 2 Department of Biological Engineering, Konkuk University, Seoul 05029, Republic of Korea

**Keywords:** antimicrobial activity, silver nanoparticles, probiotic bacteria, antioxidant, wound-healing activity

## Abstract

The prospective application of probiotics is an adjuvant for the advancement of novel antimicrobial and wound-healing agents. Currently, probiotic bacteria are utilized for the biosynthesis of nanoparticles in the development of innovative therapeutics. The present study aimed at using nanoparticle-conjugated probiotic bacteria for enhanced antibacterial and wound-healing activity. In the present investigation, the probiotic bacteria were isolated from a dairy source (milk from domestic herbivores). They screened for antibacterial activity against infection-causing Gram-negative (*Pseudomonas aeruginosa* and *Escherichia coli*) and Gram-positive (*Bacillus subtilis* and *Staphylococcus aureus*) pathogens. Further, the probiotic strain with higher bactericidal activity was used to synthesize silver, selenium, and copper nanoparticles. The isolated strain was found to be *Lactiplantibacillus plantarum* and it only has the ability to synthesize silver nanoparticles. This was verified using Ultra violet-Visible (UV-Vis) spectroscopy, where the test solution turned brown and the greatest UV-Vis absorptions peaked at 425 nm. Optimization studies on the synthesis of AgNPs (silver nanoparticles) are presented and the results show that stable synthesis was obtained by using a concentration of 1mM silver nitrate (AgNO_3_) at a temperature of 37 °C with pH 8. The FTIR (Fourier transform infrared spectroscopy) study confirmed the involvement of functional groups from the cell biomass that were involved in the reduction process. Additionally, biosynthesized AgNPs showed increased antioxidant and antibacterial activities. The nano silver had a size distribution of 14 nm and was recorded with HR-TEM (high-resolution transmission electron microscopy) examination. The EDX (energy dispersive X-ray) analysis revealed 57% of silver groups found in the nanoparticle production. The biosynthesized AgNPs show significant wound-healing capabilities with 96% of wound closure (fibroblast cells) being observed through an in vitro scratch-wound assay. The cytotoxic experiments demonstrated that the biosynthesized AgNPs are not extremely hazardous to the fibroblast cells. The present study provides a new platform for the green synthesis of AgNPs using probiotic bacteria, showing significant antibacterial and wound-healing potentials against infectious pathogens.

## 1. Introduction

In recent decades, the synthesis of nanoparticles (NPs) has been explored for various biomedical applications due to their distinct physio-chemical properties compared to conventional materials [1,2]. Generally, metallic nanoparticles acquire various advantages (viz. controllable optical properties, rapid thermal response, corrosion resistance, recyclability, reduced heat loss due to doping) [3,4,5,6,7,8]. Among different metallic NPs (gold, copper, iron, titanium, and zinc), nano-silver reveals excellent potential in therapeutic applications owing to innate antimicrobial and wound-healing properties [9,10]. Presently, silver nano synthesis is accomplished through physical, chemical, and biological (green synthesis) processes. However, physical and chemical methods to produce NPs require costly devices, and reagents, elevated voltages, extreme temperatures, and toxic solvents that put away harmful remains and by-products, raising safety and health concerns for the ecosystem [11]. Bio-based green synthesis technology has earned a significant interest as an alternate, simple, fast, easily scalable, and low-cost route where NPs are synthesized through natural materials (extracts obtained from plants and microorganisms) [12,13]. These natural products have an advantage of being biocompatible as they are abundant in bioactive composites, which are agreeable to act as a reducing and doping agent [14]. The NPs synthesized from natural materials that are pervasive in metabolites are recognized for their inherent nature of antibacterial inhibition (in food borne pathogens and antibiotic resistant bacteria) [8,15] and wound-healing activity (owing to water holding ability) [16,17]. 

Bacteria cause several diseases in animals and humans often leading to persistence of infections, which may result in delayed wound healing. It is very much essential to develop a new strategy in exploring novel antibiotic agents to combat the multi-drug resistant microbial strains. The metal NPs, especially silver, are used as antimicrobial agents in controlling a variety of bacteria in human systems where they protects cuts, burns, and wounds against infections [18,19]. The majority of current wound-healing methods do not produce agreeable healing results, either anatomically or physiologically. Due to its varied physiochemical features, nanotechnology is a trustworthy study area for medicines which promote wound healing. The capability of silver being sterile has long been known and used in traditional treatments and food products. Numerous studies have examined the antimicrobial properties of silver nanoparticles [9,20]. In recent times, efforts have been made in developing biological methods for the synthesis of nanomaterials that are highly focused to explore a variety of therapeutic agents. Among them, bacterial-mediated production of silver NPs has gained attention in formulating new antibacterial and wound-healing agents.

Probiotics are the microbial flora that inhabit the human body and regulate various metabolic functions; they have been linked with several dairy goods [21]. The strains of the genera, *Lactobacillus* and *Bifidobacterium*, *Bacillus*, *Pediococcus* and some yeasts, are the commonly occurring microbial probiotic flora. They have a significant role in shielding against harmful microorganisms to make the immune system stronger against infectious diseases [22], especially in dental bone resorption lesions [23,24]. The products derived from probiotics, along with bacterial extracts, have been examined for their wound-healing and antimicrobial activities as they are supposed to relieve allergies, the common cold, and decrease the risk of colon cancer and cholesterol amounts. Probiotics are beneficial in preventing and curing respiratory tract infections brought on by viruses including the flu and syncytial viruses [25]. Most of the probiotics (bacteria, molds, or yeasts) belong to the lactic acid bacteria family and are consumed in the form of yoghurt, fermented milk, or other fermented foods [26,27]. Numerous lactic acid bacteria strains are effective probiotics that have a variety of therapeutic effects [28,29]. Previous studies have also been conducted to evaluate the use of probiotics to enhance wound healing [30,31].

Though some studies have put forth the use of probiotic bacteria, *Lactiplantibacillus plantarum* derived from wound dressings shows an improvement in promoting cutaneous wound-healing; however, the antimicrobial and wound-healing effect of nanoparticles synthesized from the probiotic *Lactiplantibacillus plantarum* is still not explored. The current study has attempted to synthesize metal nanoparticles (silver and copper) by using probiotic bacteria. The antibacterial and antioxidative activity of synthesized nanoparticles were also evaluated. The synthesized nanoparticles were further optimized and characterized with ultraviolet-visible (UV-Vis) spectroscopy, Fourier-transform infrared spectroscopy (FTIR), transmission electron microscopy (TEM), and energy dispersive X-ray analysis (EDX). The in vitro wound-healing activity of the synthesized silver nanoparticles (AgNPs) was also investigated.

## 2. Results and Discussion

### 2.1. Isolated Probiotic Bacteria

**Figure 1:**
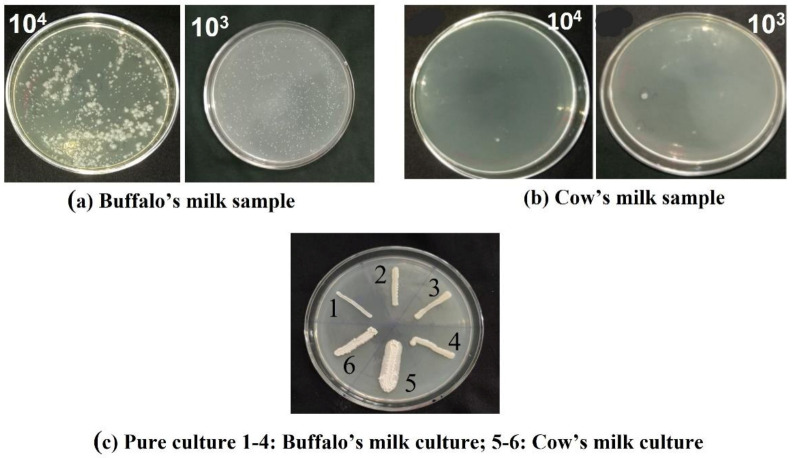
Growth of bacterial colonies cultured from 100 μL of sample of (**a**) Buffalo milk and (**b**) Cow milk from the diluted milk samples (1:10^3^ and 1:10^4^) spread over the MRS agar plates and incubated for 48 h was screened and isolated Pure culture (**c**) of bacterial colonies were recorded.

The serially diluted milk samples were spread over the Rogosa and Sharpe (MRS) agar plates and incubated for 24 h. The isolated bacterial colonies from buffalo and cow milk are shown in Figure 1a,b respectively. Six colonies were selected and re-cultured over the MRS agar plates (Pure Culture 1–4 is from buffalo milk; culture 5 and 6 were isolated from cow milk) as shown in Figure 1c.

### 2.2. Screening of Isolated Colonies

**Figure 2:**
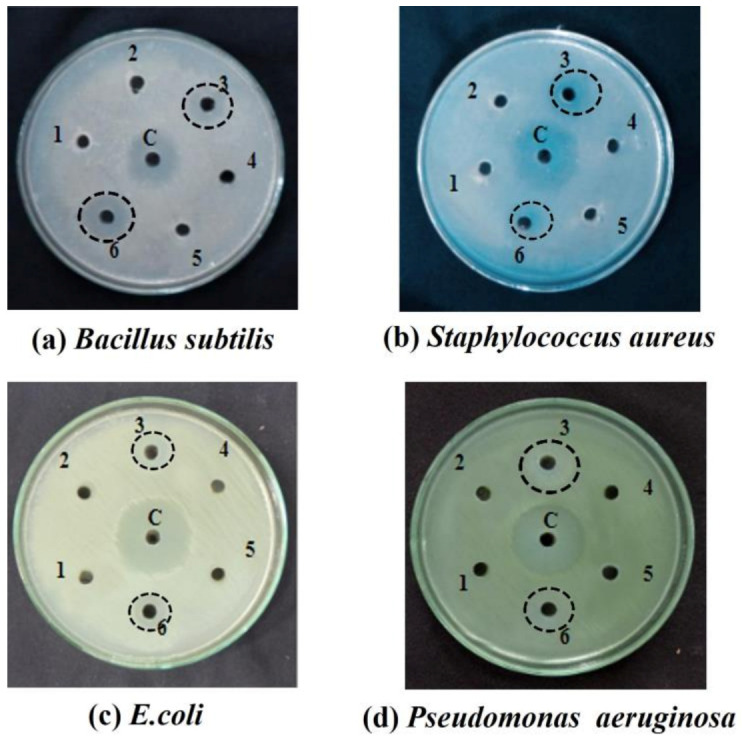
Antibacterial activity of isolated pure culture bacterial strain of buffalo milk (well no: 3) and cow milk (well no: 6) against (**a**) *Bacillus subtilis;* (**b**) *Staphylococcus aureus;* (**c**) *Escherichia coli;* (**d**) *Pseudomonas aeruginosa.*

The antimicrobial principle is especially important in probiotics, and it is one of the functional beneficial requirements of probiotics for their antagonistic effects on bacterial pathogens with a strong antimicrobial activity. Antibacterial activity against Gram-positive and Gram-negative pathogens was tested in the isolated bacterial strains. The isolated pure cultures with the highest antimicrobial property against Gram-negative *Escherichia coli* and *Pseudomonas aeruginosa* and Gram-positive *Staphylococcus aureus* and *Bacillus subtilis* were Pure Cultures 3 from buffalo milk and 6 from cow milk (Figure 2), respectively. The measured inhibition zones are tabulated in Table 1.

Evidence demonstrating the high anti-microbial activity of Pure Cultures 3 and 6 on various pathologies, caused by the release of bacteriocins, biosurfactants, H_2_O_2_, and organic acids [32], confirmed our findings. A pure culture of *Lactobacillus* species may even be antagonistic against pathogens in conditions with a lower pH (pH 6), with the synthesis of lactic acid [33].

### 2.3. Identification of Isolated Strain

The pure culture obtained from the cow’s milk in well number six was further used for molecular identification and nano synthesis. The selected probiotic bacterial strain was identified as *Lactiplantibacillus plantarum*. According to FAO/WHO (Food and Agricultural organization/World health organization) recommendations, looking for and finding probiotic microbes with 16S rRNA patterns is a viable, profitable, and acceptable process compared to other costly and time-consuming molecular approaches [34]. Using 16S rRNA primers, a sequencing investigation was carried out to determine the species of bacteria isolated from buffalo milk and uploaded to National Center for Biotechnology Information (NCBI-GenBank: ON287038.1). *Lactiplantibacillus plantarum* was discovered to be a species of bacterium that was isolated after blasting the sequence with the NCBI blast homology search tool. We used the phylogenetic tree and sequencing results of the probiotic bacteria *Lactiplantibacillus plantarum*, which are displayed in the Supplementary Figure S1a,b. Researchers discovered matching nucleotide sequences when using typical dairy sources [35].

### 2.4. Nanoparticles Synthesis

**Figure 3:**
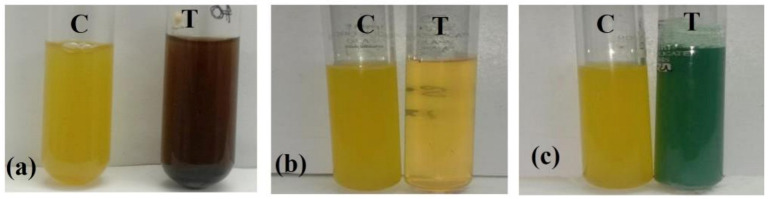
The synthesis of Nanoparticles using *Lactiplantibacillus plantarum* supernatant: (**a**) treated with silver nitrate solution which turned dark brown after 24 h, (**b**) treated with Sodium selenite which showed no color change after 48 h, (**c**) treated with Copper Sulphate which showed no color change after 24 h.

**Figure 4:**
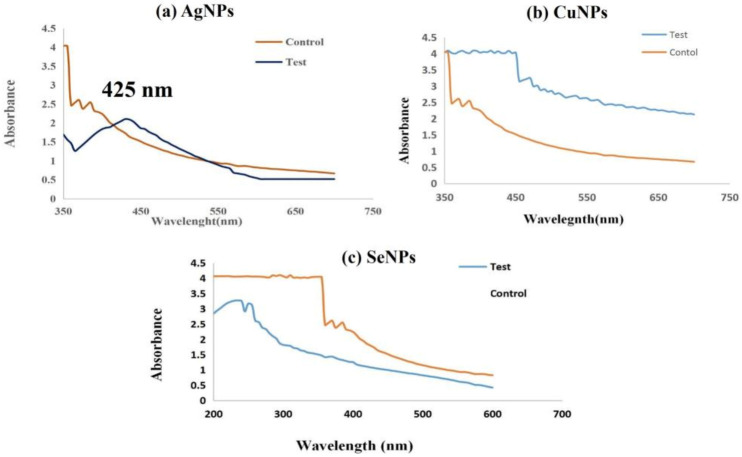
UV-Visible spectrum of synthesized silver nanoparticles (Test) showing maximum Plasmon surface peak at 425 nm (**a**). Maximum peak was not formed by copper nanoparticles (**b**) or Selenium nanoparticles (**c**).

The probiotic bacteria *Lactiplantibacillus plantarum* was further explored for its potential in the biosynthesis of metal nanoparticles. When 1 mM AgNO_3_ was added to the *Lactiplantibacillus plantarum* supernatant and incubated in dark for 24 h, the reaction mixture turned into a dark brown color (Figure 3a) which indicated the presence of silver nanoparticles (AgNPs). No color change was observed when 1mM sodium selenite (Na_2_SeO_3_) (Figure 4b), incubated in dark for 48 h, was added, nor when the bacterial supernatant was treated with 1 mM of copper sulphate (CuSO_4_) (Figure 3c), incubated in the dark for 24 h.

This was proven by the characterization study of nanoparticles also, where the reduction of Ag^+^ to AgNPs in the aqueous solution was monitored using UV-Visible spectroscopy within the range of 300 to 600 nm. AgNPs showed a strong absorbance band in the range of 410–430 nm and showed a maximum Plasmon surface peak at 425 nm and widening of the peak indicated that the particles were poly-dispersed (Figure 4a), whereas no surface plasmon peak was observed in copper and selenium nanoparticles (Figure 4b,c). Similar to this, the synthesis of AgNPs was verified in earlier research [36,37] by UV-Vis absorption from *Lactobacillus bulgaricus* at the visible range of 410 to 430 nm by the supposed peak for AgNPs.

### 2.5. Stability Analysis of AgNPs

**Figure 5:**
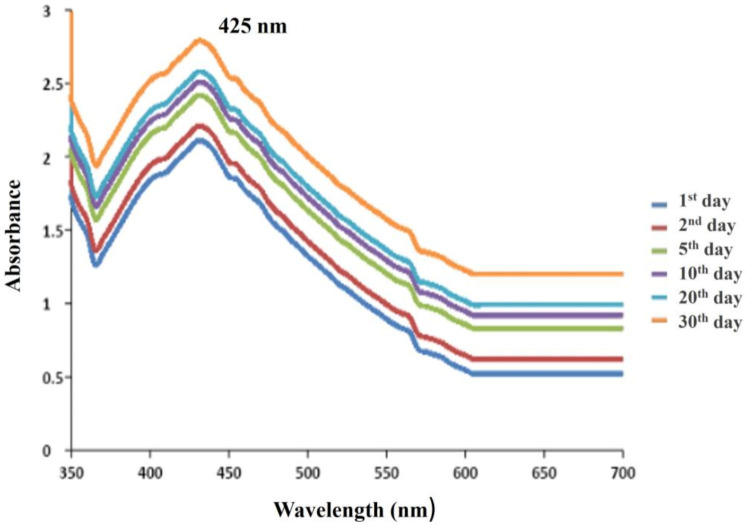
Comparison of the stability of synthesized silver nanoparticles on the 1st day, 2nd day, 5th day, 10th day, 20th day and 30th day, proving that the synthesis of nanoparticles using bacterial supernatant was a very compassionate and practical method for the synthesis of nanoparticles**.**

In order to monitor the quick decline and stability of the generated silver nanoparticles, the synthesized AgNPs were frequently examined by UV-Vis spectroscopy at varied time frames up to a month. The silver nanoparticles continued to exhibit a peak at the exact wavelength (425 nm) with the same absorption intensity even after 30 days (Figure 5). The colloidal mixture was found to be stable for 30 days, proving that the synthesis of nanoparticles using bacterial supernatant was a very compassionate and practical method for the synthesis of nanoparticles. The particles initially showed a broad peak, which suggested clusters of variable size at the initial growth stages. This is also corroborated by an earlier study, which showed that the AgNPs in the solution were stable even one month after their creation by looking at the UV-Vis spectra of milk-mediated nanoparticle samples with a peak value of 425 nm [38].

### 2.6. FTIR Analysis of AgNPs

**Figure 6:**
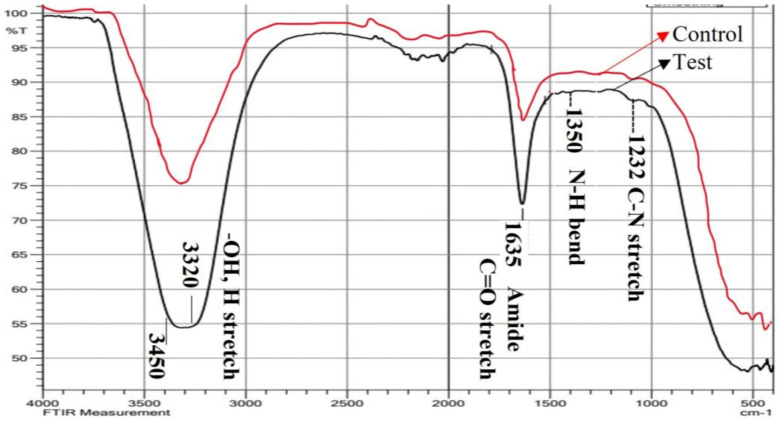
FTIR spectrum of bacterial supernatant-synthesized silver nanoparticles (test sample) in comparison with control (bacterial supernatant). Quite a few peaks denoting the functional groups of biologically active compounds were obtained in a range of 4000–400 cm^−1^ on a spectrometer.

The sample containing silver nanoparticles (test sample) and the control (bacterial supernatant) were analyzed with the Fourier Transform Infrared Spectrometer. The spectrum was recorded in AT modes with resolution 0.2 in the wavelength range of 4000–400 cm^−1^ (Figure 6). The putative functional group in the supernatant *L. plantarum* that is responsible for the reduction and stabilization of AgNPs was found using FTIR analysis of the test sample in comparison with control.

Bands at 3320 cm^−1^ found in the FT-IR spectra (Figure 6, Table 2) of silver nanoparticles produced by bacterial supernatant and control (bacterial supernatant) were attributed to the hydroxyl group and H-bonded OH stretching vibrations [39,40]. Additionally, a high concentration of proline residues was thought to be the cause of the band at 1635 cm^−1^, which showed the protein structure to be disordered [41,42]. Protein amides I and II are thought to be responsible for the conspicuous bands of about 1635 cm^−1^ and 1557 cm^−1^, respectively [43,44]. The C-N stretching (amide II) and phenyl nucleus both contributed to the absorbance at region 1350 cm^−1^, which was caused by the N-H bending [41]. As a result, free amine groups or the carboxylate ion of the amino acid residue were used to bind the proteins to the AgNPs. The milk’s -COOH group was attached to the AgNPs, according to the C=O stretching band. As a result, the spectrum at 1232 cm^−1^ IR suggested that AgNPs may be attached to proteins via free amine groups. These substances may have coupled with the Ag surface and produced the highly stable AgNPs. In order to prevent agglomeration and improve stabilization in the media, proteins essentially build a cloak around the AgNPs. Through FTIR measurements, earlier researchers [45,46] verified the existence of proteins in the produced NPs. The inclusion of biomolecules from the *L. plantarum* cell-free supernatant, which were implicated in the biosynthesis of AgNPs, was confirmed by the functional groups (hydroxyl, protein, and carboxyl) that were acquired on the biosynthesized AgNPs in this work [47,48].

### 2.7. TEM and EDX Analysis of AgNPs

**Figure 7:**
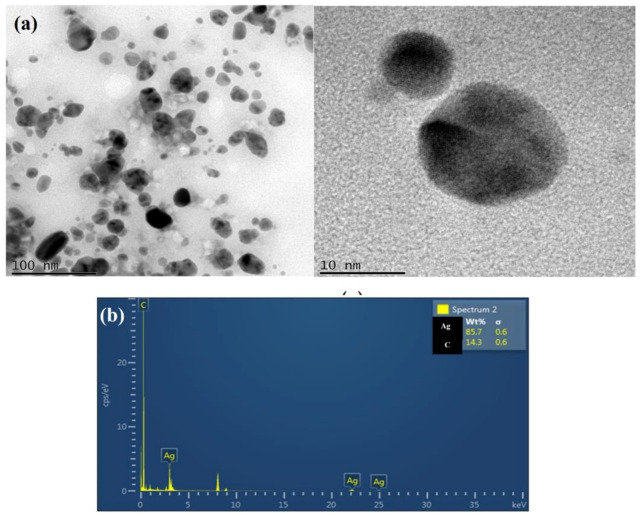
(**a**) Transmission electron microscopic examination of bacterial supernatant-synthesized silver nanoparticles shows the nano size (14 ± 4.7 nm) with sphere-shaped AgNPs synthesized from probiotics *L. plantarum*. (**b**) Energy-dispersive X-ray (EDX) spectrum of synthesized nanoparticle confirms the presence 85.7% of Silver (Ag).

The shape and size variation of biosynthesized AgNPs were examined using TEM analysis. Figure 7 shows that most of the particles are spherical, have a mean particle dimensional distribution, and are 14.0 ± 4.7 nm in size on average. Moreover, the biosynthesized AgNPs were well-dispersed, without significant agglomeration. The functional groups and protein present on the cell membrane of *L. plantarum* facilitated the formation of NPs by serving as a binding site for metal ions before the development of NPs [49]. In addition, the extracellular formation of AgNPs was confirmed by EDX spectra, which revealed an elemental silver peak implying the existence of 85.7% of silver in the synthesized nanoparticles; the other elemental peak signals in the EDX spectra are due to the chemical used for sample processing (Figure 7a,b).

### 2.8. Optimisation of Synthesised Nanoparticle

#### 2.8.1. Effect of Temperature

**Figure 8:**
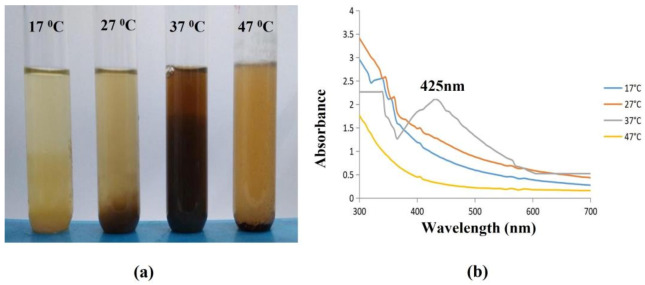
Effect of Temperature on synthesised AgNPs. (**a**) Darkening of color indicating the synthesis of AgNPs was observed at 37 °C. (**b**) The synthesis of AgNPs (37 °C) was confirmed with a spectral peak at 425 nm using a UV-Vis Spectrophotometer.

The AgNPs were highly synthesised at a temperature of 37 °C with dark brown color formation, where there was no synthesis at 17 °C, with no color change, and slow synthesis at 27 °C and 47 °C with a slight color change, which indicated the poor synthesis of AgNPs (Figure 8a).

The primary physical factor that influences the creation of NPs along with their size and shape is temperature. Examining the UV-Vis spectra at four different temperatures (17 °C, 27 °C, 37 °C, and 47 °C) confirmed this. The results demonstrated that as the temperature increased towards 37 °C, the surface plasmon resonance (SPR) spikes (at a wavelength around 425 nm) sharpened, suggesting that this temperature is ideal for biosynthesis. Conversely, as the temperature declined, the absorption peaks shrank (Figure 8b). At the ideal temperature (25 °C), the smallest particles developed, however, at lower and higher temperatures, larger NPs and aggregates tended to form [49].

#### 2.8.2. Effect of Concentration

**Figure 9:**
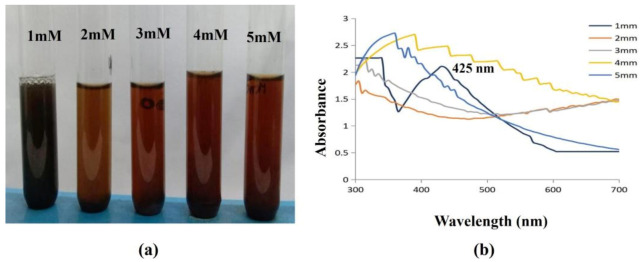
Effect of Concentration on synthesised AgNPs. (**a**) The synthesis of AgNPs was observed as a color shift towards red, indicating that the particle size gradually increases with concentration; (**b**) 1 mM concentration of AgNO_3-_synthesized AgNPs, which was confirmed with a spectral peak at 425 nm.

The UV-Vis spectra were recorded for various concentrations (1, 2, 3, 4, 5 mM) of silver nitrate solution. From Figure 9, it is evident that the peak intensity increases with the increase in concentration. This shows that the nanoparticle density increases with concentration. 

Furthermore, the wavelength peak was gradually shifted towards red with respect to the concentration. The shift towards red indicates that the particle size gradually increases with concentration. The curve sharpness also increased with concentrations, and this may be due to the formation of spherical and cubical nanoparticles. The salt concentration employed for the bio synthesis of NPs is a crucial factor in NP production optimization. A previous study discovered that the optimal concentration of AgNO_3_ needed to produce AgNPs was 0.0016 mol L^−1^ out of the several AgNO_3_ concentrations they investigated (0.0004, 0.0008, 0.0012, 0.0016 and 0.0024 mol L^−1^) [50]. In the current investigation, it was discovered that the nanoparticle synthesis of Ag was more effective when AgNO_3_ was used at a concentration of 1 mM. The findings completely agree with those made public by earlier scientists [51].

#### 2.8.3. Effect of pH

**Figure 10:**
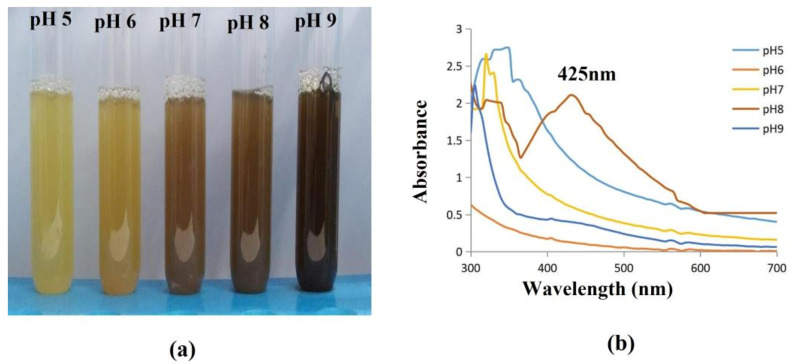
Effect of pH on synthesised AgNPs. (**a**) The AgNP synthesis was observed as a color shift towards brown indicating that the particle size gradually increases with concentration (**b**). At pH 8, stable AgNPs were formed, which was confirmed with a spectral peak at 425 nm; the solutions with a pH of 5, 6, 7, 9 showed unstable peaks.

The pH level of the solution is another crucial factor that influences how nanoparticles are formed. Since pH has the power to change the charge of biological molecules, which may have an impact on both their capping and stabilizing properties, it has an impact on the size and form of the particles. Figure 10 illustrates how the pH of the solution affects the maximal absorption wavelength and intensity. The absorption maximum moved from 300 to 425 nm when the pH was risen from 5 to 9. Along with the spectroscopic shift, the strength of the absorption rises as pH rises. At pH 8, a steady peak appeared at 425 nm, showing that this is the optimal pH for the formation of AgNPs from bacterial supernatant. When the particles were held overnight, however, at a high pH of 9, with enhanced absorbance, they were unstable and agglomerated. This was verified by prior research [52], which found that stable AgNPs were generated at a pH of 8, and that the absorbency of AgNPs derived from olive leaf extract increases as the solution pH rises from 2 to 9.

#### 2.8.4. Antibacterial Activity of AgNPs

**Figure 11:**
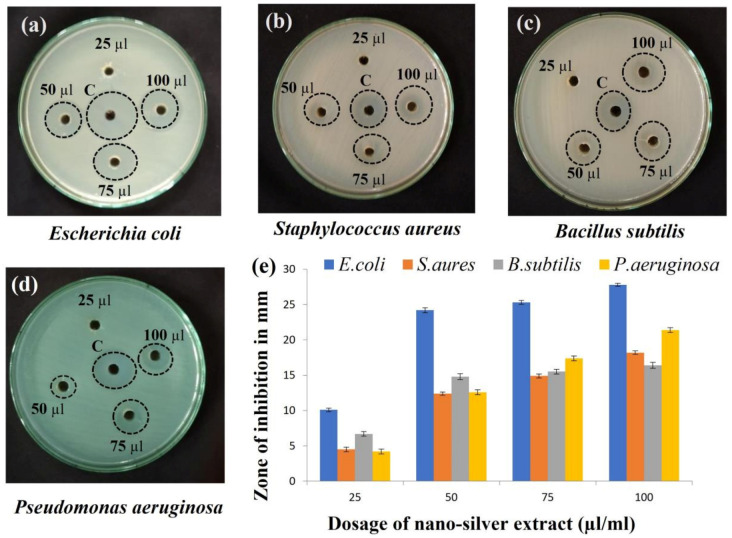
Antibacterial assay of synthesized silver nanoparticles against (**a**) *Escherichia coli* treated with bio-synthesized AgNPs, showing maximum antimicrobial activity when compared to (**b**) *Staphylococcus aureus;* (**c**) *Bacillus subtilis;* (**d**) *Pseudomonas aeruginosa.* Zone of inhibition with different dosage of nanosilver treatments are represented by bar graphs (**e**). Standard deviation (±) shows the mean values of 3 replications. The results are statistically significant with *p* value < 0.05.

Gram-negative (*Escherichia coli* and *Pseudomonas aeruginosa*) and Gram-positive (*Staphylococcus aureus* and *Bacillus subtilis*) bacterial growth were inhibited by the AgNPs and was observed by the zone of inhibition. Moreover, when different volumes of AgNPs (25, 50, 75, and 100 µL) were used for the zone of inhibition study (Figure 11), the zone of inhibition expanded as the concentration did. Between 10 and 15 nm in size, silver ions doped with metabolites have improved stability, biocompatibility, and antibacterial activity [53]. This was seen in the current work as well, where the generated silver nanoparticles were 14.0 ± 4.7 nm in size on average. As a result of Ag ions with metabolites attaching to cell membranes and altering the lipid bilayer, cells become more permeable, suffer damage, and eventually die [54]. This powerful antibacterial action appears to be more prominent when relatively smaller nanoparticles are utilized.

The adhesion and accretion of AgNPs on the cell surface were especially observed for Gram-negative bacteria. The outer layer of Gram-negative bacteria contains water-filled channels known as porins that AgNPs can pierce all the way through. Porins play a major role in the passive translocation of hydrophilic molecules of different sizes and charges across membranes. The action of AgNPs was more pronounced in Gram-negative bacteria than in Gram-positive bacteria, probably as a result of the stiffer cell wall of Gram-positive bacteria causing the passage of silver ions into the cytoplasm [55]. It is also plausible that the presence of lipopolysaccharides increases the morphological integrity of the cell wall of Gram-negative bacteria, rendering these bacteria more susceptible to silver nanoparticles, as the lipopolysaccharides’ negative charge promotes AgNP adherence [56]. The efficacy of the metabolite present in the supernatant along with silver ions appended to the bacterial cell wall, owing to the electrostatic interaction among positively charged silver ions and the negatively charged surface of the cell membrane because of the phosphate, carboxyl, and amino groups, provide an occasion to consequently go through it, thereby causing structural modification in the cell membrane and affecting its permeability. Then, dissipation of proton motive force (PMF) occurs, and, thus, membrane damage occurs [57,58]. AgNPs possibly also act as a transporter to transport Ag^+^ more resourcefully to bacterial cells whose proton motive force would therefore reduce the local pH and increase Ag^+^ release [59]. Adding the evidence together, it is supposed that silver nanoparticles form free radicals upon interacting with bacteria that break the cell membrane, making it porous [60].

#### 2.8.5. Antioxidant Activity

The pathophysiology of chronic inflammatory disorders is linked to oxidative stress, which is brought on by tissues with excessive amounts of reactive oxygen species (ROS). The development of cellular oxidative stress in microorganisms is also a marker of the harmful effects of heavy metal ions such as Ag^(+)^. As a result, an upsurge in cell oxidative stress can be predicted to be caused by an enlarged proportion of Ag ^(+)^ ions. AgNPs’ potent antibacterial action is a result of their ability to produce ROS and free radical species, including singlet oxygen, hypochlorous acid, hydroxyl radicals, and hydrogen peroxide (H_2_O_2_), superoxide anion, and hydroxyl radical (OH∙) [61].

**Figure 12:**
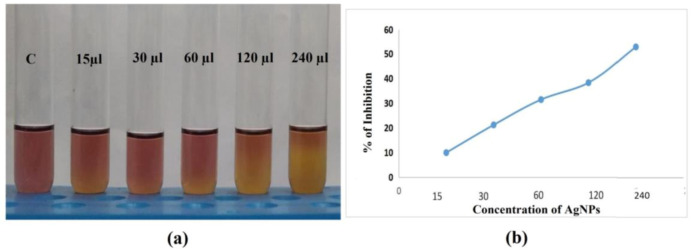
Antioxidant activity of synthesized Silver Nanoparticles of different concentrations. (**a**) The results of 0 µL (control C), 15 µL, 30 µL, 60 µL, 120 µL, and 240 µL concentrations, as measured by the DPPH assay, formed a linear graph (**b**), proving that as the concentration of AgNp increases, scavenging activity also increases from 9.9 to 52.4%.

The antioxidant action of AgNPs was measured by the DPPH (2,2-diphenyl-1-picryl- hydrazyl-hydrate) assay. The antioxidants’ en-1, 2-diol and dien-1, 4-diole moieties can decrease the DPPH radical. Figure 12a illustrates the antioxidant properties of the AgNPs towards DPPH and demonstrates how the antioxidant impact is related to the removal of DPPH in the sample material. The scavenging activity slowly and linearly increases with the increase in concentration of supernatant with silver ions (15–240 µL/mL) (Figure 12b). The supernatant with silver ions showed scavenging activity ranging from 9.9–52.4%. Similar results were obtained in several other studies against DPPH. The significant antioxidant capacity of the nanoparticles has been linked to various organic compounds derived from spice mixtures, which are thought to be responsible for lowering and encapsulating the Ag ions, according to some authors [62,63].

### 2.9. Cytotoxicity of AgNPs

**Figure 13:**
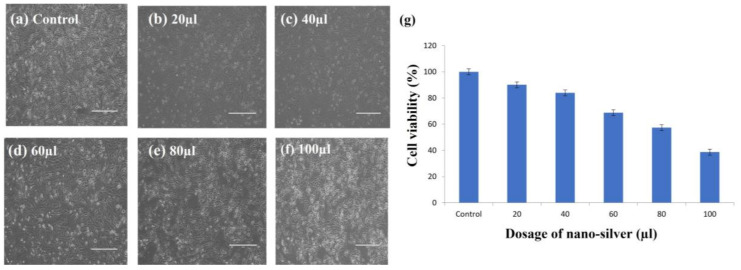
MTT assay of bio-synthesized AgNPs against 3T3 cells (**a**) at different concentrations of AgNPs. **a**. Control, **b**. 20 µL, **c**. 40 µL, **d**.60 µL, **e**. 80 µL, **f**. 100 µL proved to decrease the survivability of the cells (**g**) with an increase in the concentration of synthesized AgNPs. The size measurement of scale bar line is 500 µm (**a**–**f**). Experiments were repeated thrice and (**g**) the graph indicates the percent of cell viability at different dosage of nano-silver treatments. The error bar presented as mean ± SD which are statistically significant with *p* value < 0.05.

The amount of ATP in the cell is significantly decreased by silver nanoparticles, which ultimately damages the mitochondria and promotes the production of reactive oxygen species (ROS) in something similar to a dose-dependent manner. Therefore, the MTT assay was used to determine the lethality of AgNPs at various doses (20, 40, 60, 80, and 100 µL/mL) (Figure 13), and the density of cells was measured using inverted microscopy after 24 h.

Silver nanoparticles (AgNPs) offer promising applications in the treatment of breast cancer, skin cancer, and open wounds [64]. Additionally, they are utilized as prosthetic materials and bone cement for quick healing. The MTT test was used to calculate the viability (%) of the 3T3 cell line following treatment with biosynthesized silver nanoparticles. Figure 13 displays the survivability of the cells (%) exposed to different AgNP concentrations. The 3T3 cell viability was significantly reduced when treated with 80–100 µL of silver nanoparticles, according to the results, which showed that the biologically synthesized AgNPs had moderate to low cytotoxicity. From the results it was found that the biosynthesized silver nanoparticles are not highly toxic to the fibroblast cells. In the earlier study, the proteomic data on AgNP-treated microbial cells showed an accumulation of immature membrane precursor proteins that cause destabilization of the outer membrane of microorganisms such as *E. coli* [65] and *Staphylococcus aureus* [66]. The building up of undeveloped precursor proteins promotes the attenuation of proton motive forces and the sharp decline of cellular ATP production, the latter of which may be caused by seepage or the downregulation of ATP synthesis [61,67,68]. This is because the migration of precursor proteins to the cell membrane requires energy from both ATP and proton motive forces.

The AgNPs also exhibit a toxic effect on macrophages, especially those with the smallest particle sizes. Researchers [69] who used the four cell lines A549, HepG2, MCF-7, and SGC-7901 discovered that AgNPs (15 nm) exhibited the maximum cytotoxicity as predicted, supporting this claim. According to research by another scientific team [70], 5 nm AgNPs are more hazardous than both 20 and 50 nm AgNPs. According to reports, cytotoxic characteristics are significantly influenced by the form of nanoparticles. Depending on the synthesis circumstances, AgNPs can be generated in a variety of forms [71,72,73]. AgNPs thus display bactericidal effectiveness that is dependent on form. For instance, whereas wires caused poor results, aggregates did not exhibit negative impacts on cytotoxic measures in A549 cells [74]. According to another study, AgNPs with identical surface areas but various shapes had variable antibacterial effects. In comparison to spherical and rod-shaped nanoparticles, they discovered those truncated triangle silver nanoplates with a lattice plane, such as a basal plane, showed the best biocidal effect [57]. Biosynthetic AgNPs in the current work have a range of size comprising 14 ± 4.7 nm and are spherical. They have a moderate cytotoxic effect against fibroblast cells.

### 2.10. In Vitro Scratch Wound-Healing Activity of AgNPs

**Figure 14:**
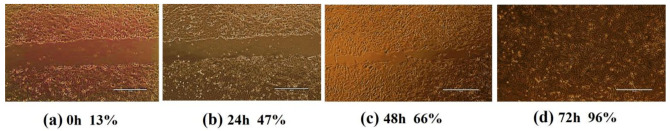
In vitro scratch wound-healing activity of AgNPs. (**a**) At the initial time, 0 h, this showed 13% cell migration in wounded scratches; (**b**) at 24 h, wound-healing activity started to increase with 47% cell migration; (**c**) at 48 h, it increased to 66%; (**d**) at 72 h, a wound-healing efficacy of 96% was observed under an inverted microscope. The scale bar is 500 µm (**a**–**d**).

The scratched Vero cells were exposed to 60 μL/mL biosynthesized AgNPs at different times: 24, 48, 72 h at 37 °C. The treated cells after periodic incubation were observed and analyzed for their wound recovery and growth of the fibroblast cells. The injury-curing efficacy of the biosynthesized AgNPs and the non-treated cells were observed under an inverted microscope and the percentage of wound scarring was computed. After 24 h of exposure to the AgNPs, cell migration was at 47% in wounded cells, then after 48 h, the injuries were cured to an efficacy of 56.58% with the biosynthesized AgNPs, and after 72 h, a wound-healing efficacy of 96% was observed. The following Figure 14 reveals that the effect of an increase in the AgNP exposure period increased the cell migration in fibroblast 3T3 cells.

After scratching the cells, the rise in the cell mass can be due to the active proliferation and migration of fibroblastic cells towards the injured region. It points toward the successful wound-healing action of synthesized AgNPs. The contractile elements appearing in the fibroblastic cells may be provoked by the conversion of fibroblasts to the myofibroblasts and amplified passage of cells. The fibroblasts’ differentiation to myofibroblasts and the promotion of wound contraction might be led by AgNPs. The structure of the healed cells looked alike to normal cells. This confirms that AgNPs notably have high-quality healing capacities. It was stated by the earlier reports that the antimicrobial and antioxidant actions have positive results on wound healing along with cell migration and proliferation, which plays a crucial role in the healing process. This was found to initiate the proliferative phase of repair. In the present period, the biomedical product that is coated with AgNPs was typically employed to promote faster wound healing and prevent microbiological infections. AgNPs also could influence cytokine production and possess anti-inflammatory properties [75]. The extract of bioAgNP–propolis-coated sutures enhanced the growth of 3T3 fibroblasts in an early in vitro scratch experiment for wound healing [76]. The current study has demonstrated that silver nanoparticles made with the probiotic bacteria *Lactiplantibacillus plantarum* have greater antimicrobial activity against pathogens that induce wound infections as well as effective in vitro wound-healing activity against fibroblast (3T3) cell lines (obtained from NCCS, Pune, India) after 72 h. This proves that the AgNPs produced from *Lactiplantibacillus plantarum* possess antimicrobial and anti-inflammation properties.

## 3. Materials and Methods:

### 3.1. Isolation of Probiotic from Milk

**Figure 15:**
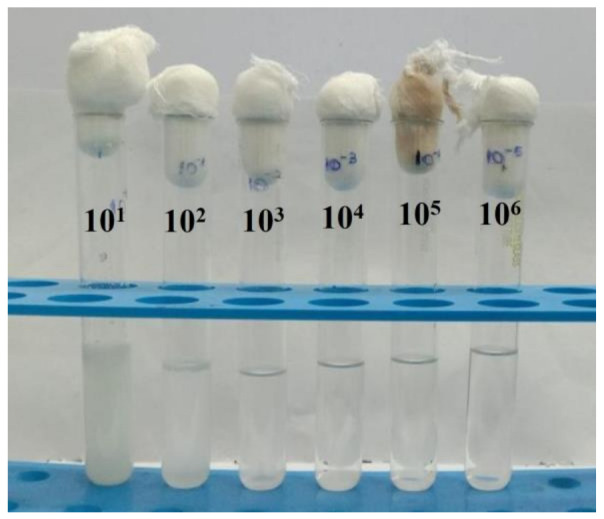
Milk samples (cow and buffalo milk) serially diluted ranging from 10^−1^–10^−6^.

The milk samples from cow and buffalo (50 mL) were collected from a local area of Kanchipuram, Tamandu, India and stored in a sterile environment. Chemicals of analytical grade (SRL chemicals, Chennai, India) were used for isolation of probiotics from milk in the present study. About 5.5 g of De Man, Rogosa and Sharpe agar (MRS) and 20 g of agar was added to 100mL of distilled water. The milk samples were serially diluted ranging from 10^−1^–10^−6^ (Figure 15). A total of 100 µL of sample from the 10^−3^ and 10^−4^ dilutions was spread over the MRS agar plates and incubated at 36 °C for 48 h [77]. 

Six colonies were selected and isolated from the plates and re-cultured on the MRS agar. The selected six colonies were screened for antibacterial activity against infection-causing pathogens using the agar well diffusion method [78]. The experiments were performed with 3 replicates.

- Once swabbed over LB media, they were screened against Gram-positive *Bacillus subtilis* and *Staphylococcus aureus*, as well as Gram-negative *Pseudomonas aeruginosa* and *Escherichia coli*.
- To the freshly prepared MRS broth a loop full of *Lactobacillus* culture was added and incubated in shaker for 48 h.
- Mueller-Hinton agar plates were prepared with 100 μL of infection-causing pathogens (Gram-positive *Bacillus subtilis* and *Staphylococcus aureus,* as well as Gram-negative *Pseudomonas aeruginosa* and *Escherichia coli*) spread evenly onto the surfaces of the agar plates separately.
- The MRS broth containing *Lactobacillus* culture was centrifuged and 50 µL of supernatant was added to the wells. Gentamycin (MEDOX Biotech, Chennai, India) was used as the control and the plates were incubated for 24 h.

### 3.2. Identification of Probiotic Bacteria

The pure culture obtained from the cow’s milk in well number 6 was further used for molecular identification and nano synthesis. The 16S rRNA sequencing method was utilized to identify the isolated bacterial strain [79]. Polymerase chain reaction (PCR) kit (MEDOX Biotech) was used for the study. Initially, PCR was used to isolate and amplify the genomic DNA of the isolated strain of bacteria using various enzymes and primers. For genomic DNA amplification, the forward primer (27F-5’ AGAGTTTGATCMTGGCTCAG 3’) and the reverse primer (ITS4-5’ TACGGYTACCTTGTTACGACTT’1’3’) were utilized. The amplified genomic DNA was isolated from the PCR mixes after 25 PCR cycles. At YaazhXenomics, Coimbatore, India, single-pass sequencing was performed using 16S rRNA universal primers in an ABI 3730xl sequencer. The phylogenetic tree for the sequenced sample was created by means of the PhyML 3.0 tool (available in the online) using multiple sequence alignment, phylogeny analysis of the query sequence with their closely related sequence of blast, and multiple sequence alignment.

### 3.3. Synthesis of Nanoparticles

The selected probiotic bacterial strain was identified as *Lactiplantibacillus plantarum* and utilized as a reductant in the biosynthesis of metal NPs. The isolated bacterial strain (*Lactiplantibacillus plantarum*) was grown in 100 mL of MRS broth for 24 h at room temperature. Then, the culture was centrifuged at 8000 rpm for 10 min, the pellet was discarded, and the supernatant was used for the nanoparticles’ synthesis. Three metals were used for the nanoparticle synthesis (silver, copper and selenium), along with the supernatant of the microbial culture and those nanoparticles which formed a stable peak when tested with a UV spectrophotometer (Hitachi U2900, Tokyo, Japan), which was used for further analysis (300–700 nm). A total of 20 µL of 1 mM silver nitrate (AgNO_3_) was added to the 5ml of supernatant and the reaction mixture was kept undisturbed in dark at room temperature for 24 h [80]. Then, 1mL of 5 mM copper sulphate (CuSO_4_) was added to the 5 mL of supernatant and incubated in dark at room temperature for 24 h [81,82]. The reaction mixtures at a concentration of 5 mM, 10 mM and 15 mM were made by adding sodium selenite (Na_2_SeO_3_) to the 5 mL of supernatant, and were incubated in the dark at room temperature for 48 h [83].

### 3.4. Optimization of Nanoparticles Synthesis

The cell-free supernatant obtained from the bacterial strain (*Lactiplantibacillus plantarum*) was used in the preparation of reaction mixtures. The optimization process of the nano synthesis was carried out using one factor at a time (OFAT).

#### 3.4.1. Effect of Temperature

The produced reaction mixtures were maintained at various temperatures (17 °C, 27 °C, 37 °C, and 47 °C) to examine the impact of temperature on the synthesis of AgNPs. At each temperature range, the aqueous colloidal suspensions’ electronic absorption spectra were captured.

#### 3.4.2. Effect of pH

Initially, the pH of the reactant was 6. Using (0.1 N) HCl and (0.1 N) NaOH, the pH of the reaction mixture was kept at 5, 6, 7, and 8 points, respectively. The resulting solutions’ absorbance was calculated spectrophotometrically using the Hitachi U2900 spectrophotometer (Hitachi, Tokyo, Japan) within the wavelength region of 300–700 nm.

#### 3.4.3. Effect of Precursor Concentration

The reaction mixtures were generated at various concentrations, including 1 mM, 2 mM, 3 mM, 4 mM, and 5 mM, to evaluate the impact of concentration on the AgNPs production. The reaction mixtures’ absorption spectra were then spectrophotometrically recorded using the Hitachi U2900 spectrophotometer (Hitachi, Japan) within the wavelength region of 300–700 nm.

### 3.5. Biosynthesis and Stability Analysis of Synthesized Nanoparticles

**Figure 16:**
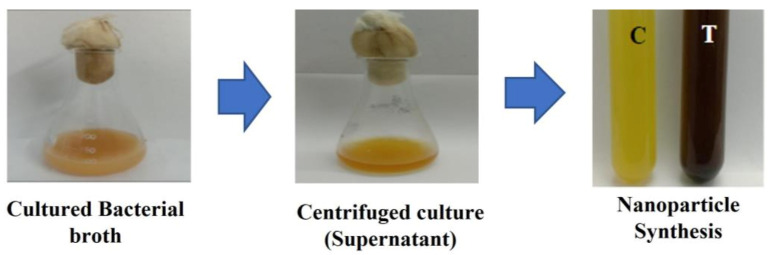
Biosynthesis of Nanoparticles.

Analytical-grade chemical reagents were used in this experiment. The bacterial supernatants (T) were treated with AgNO_3,_ CuSO4, Na_2_SeO_3_, and the control (C), which was also maintained simultaneously (Figure 16). Using a UV-visible spectrophotometer (Hitachi U2900, Japan) in the optical range of 300 to 700 nm, the depletion of metal ions in the water-based solution was observed. A change in color of the aqueous solution with metal ions were observed for the confirmation of synthesized nanoparticles [80,81,82,83].

In order to monitor the quick reduction and stability of generated silver nanoparticles, the prepared formulations were frequently examined by UV-Vis spectroscopy at varied time intervals up to a month [84,85].

### 3.6. Characterization of Nano Particles

The AgNPs were characterized by a variety of techniques, such as UV-Visible spectroscopy (U2900, Hitachi, Tokyo, Japan), and transmission electron microscopy (TEM) (FEI Tecnai, Hillsboro, OR, USA) equipped with an energy-dispersive X-ray (EDX). The TEM analysis was performed to investigate the morphology and size distribution of biosynthesized AgNPs. In addition, EDAX analysis was used to determine whether silver was present in the synthesized particles [86], and Fourier transform infrared spectroscopy (FT-IR) was used to determine whether functional groups were present. The analysis of dried pellets of synthesized silver nanoparticles was performed using a Perkin Elmer Fourier Transform Infra-Red (FTIR) Spectroscopy C100599 Instrument, which has a resolution of 0.4 cm^−1^ at the working range of 4000–400 cm^−1^ [48].

### 3.7. Application Studies

#### 3.7.1. Antibacterial Assay of Bio-Synthesized Nanoparticles

The antibacterial assessment of bio synthesized nanoparticles was performed by the agar well diffusion method against Gram-positive (*Staphylococcus aureus* and *Bacillus subtilis*) and Gram-negative (*Escherichia coli* and *Pseudomonas aeruginosa*) pathogens. The inoculum of each pathogen was grown overnight in nutrient broth at 37 °C. Sterilized swabs were used to streak each pathogen onto the nutrient agar. Simultaneously, wells were punched with a sterile borer and nanoparticles were added to the well at different concentrations (25, 50, 75 and 100 µL). The plate was incubated at 37 °C for 24 h, and the diameter of the inhibition zone was measured [87,88]. The experimental data were recorded from the mean of three replicates.

#### 3.7.2. Antioxidant Activity of Bio Synthesized Nanoparticles

The antioxidant activity of bio synthesized nanoparticles was determined by the 2,2- diphenyl-1-picrylhydrazyl (DPPH, purchased from Himedia Lab. Pvt. Ltd., Mumbai, India) free radical scavenging assay (RSA) [78]. Briefly 2 mL of 0.1 mM DPPH (3.94 mg dissolved in 100 mL methanol) was added a to 2 mL aliquot of different concentrations (15.6, 31.3, 62.5, 125, 50 µL/mg) of bio synthesized nanoparticles. The mixture was vortexed thoroughly and incubated in the dark for 30 min. The absorbance was recorded at 517 nm using UV-Visible spectroscopy at the end of the incubation. DPPH without the sample was used as the control, and methanol was used as the blank. We then calculated the percentage inhibition using the formula

$$\%\;\mathrm{Inhibition}=\frac{\left(\mathrm{Absorbance}\;\mathrm{DPPH}\;\mathrm{control}\;-\;\mathrm{Absorbance}\;\mathrm{DPPH}\;\mathrm{sample}\right)}{\mathrm{Absorbance}\;\mathrm{DPPH}\;\mathrm{control}}\;\text{×}\;100$$

#### 3.7.3. In Vitro Wound-healing Activity

##### Cell Culture Maintenance

The National Centre for Cell Sciences (NCCS), located in Pune, India, provided the 3T3 cells (normal fibroblast cell line) for study. In Dulbecco’s modified eagle medium (DMEM), which has been supplemented with 10% (*v*/*v*) heat-inactivated fetal bovine serum (FBS), 100 g/mL streptomycin, and 100 U/mL penicillin cells were kept in the logarithmic phase of growth [89]. They were kept in a 95% air humidified incubator at 37 °C with 5% CO_2_.

##### Cytotoxic Effect

By using the MTT (3-(4,5-dimethylthiazol-2-yl)-2,5-diphenyltetrazolium bromide assay kit (from MEDOX Biotech), the cytotoxic effect of the AgNPs was evaluated against the 3T3 cell line [90]. The cells were plated in 96-well microplates at a density of 1 × 106 cells per well, and then cultured at 37 °C for 48 h in a 5% CO_2_ incubator to reach 70–80% confluence (3 replicates were maintained for data collection). The media were then changed, and the cells were exposed to various doses of AgNPs while being incubated for 24 h. After 24 h, the morphological alterations of the untreated (control) and treated cells were examined and documented using a digital inverted microscope (20X magnification). After using phosphate-buffered saline (PBS, pH 7.4) to wash the cells, 20 µL of the (MTT) solution (5 mg/mL in PBS) was applied to each well. After that, the plates were left at 37 °C in the dark for two hours. The absorbance was measured spectrophotometrically at 570 nm after the formazan crystals were dissolved in 100 L DMSO. The amount of cell viability was calculated using the following formula,

Cell viability (%) = (Absorbance of sample/Absorbance of control) × 100.

The concentration of the analyte was displayed on the X-axis, with the cell viability (%) on the Y-axis.

#### 3.7.4. In Vitro Scratch Wound-Healing Assay

The previously described and established protocol was followed for this experiment [91]. In the ideal culture conditions, Vero cells were sown in 6-well plates (8 × 10^5^ cells/well) and cultivated until they attained a confluence of 90–95%. A P10 micropipette was used to create a scratch in the center of the cell monolayer to simulate a wound. The cells were then washed with water to remove cell debris with fresh media. Mouthwash sample (50 g/mL) was applied to the wound and left on for 24–48 h at 37 °C in a humidified incubator with 5% CO_2_. The cells serving as the negative controls were kept untreated (three replicates were maintained for data collection). Two modalities were used to investigate the scratch-healing process.

We captured digital pictures at time 0 (T0), 24 h (T1), and 48 h (T2) using the digital inverted microscope (static imaging). Using Image J processing software, the distance between the wounded widths at T0 and T1/T2 was used to quantify the scratch’s closure. The formula used to get the scratch closure rate (SCR), as published by earlier researchers [92], is as follows:

SCR = ((T0 − T1/T2)/T0) × 100

where, T0 is the scratch area at time 0 and T1/T2 is the scratch area at 24 h and 48 h.

## 4. Conclusions

On a concluding note, the present study demonstrated that raw buffalo milk is one of the best sources for the isolation of probiotic bacteria. The supernatant was successfully created for the bacteria-mediated production of AgNPs. This is the first study where *Lactiplantibacillus plantarum* was used to synthesize AgNPs, and it was revealed that they were stable for a month. Additionally, compared to other research, the standard area of the NPs produced in the current work is lower (14.0 ± 4.7 nm). As a limiting and encapsulating tool for the fabrication of stable and functionalized AgNPs, bacterial biomolecules have been crucial. The biosynthesized AgNPs demonstrated increased wound-healing abilities as well as potential bactericidal activities against human infections. The utilization of AgNPs for the management of infectious pathogens and wounds is justifiable in light of these promising therapeutic qualities. Further clarification of the mechanism behind the antibacterial inhibitory and wound-healing effects of biosynthesized AgNPs is also required.

## Figures and Tables

**Table 1:** Zone of inhibition (in diameter) due to isolated pure cultures of bacterial strains against Gram-negative bacteria (*Escherichia coli* and *Pseudomonas aeruginosa*) and Gram-positive bacteria (*Staphylococcus aureus* and *Bacillus subtilis*). The experimental data were collected in triplicate.

Microorganism		Zone of Inhibition (mm)		
		Positive Control&&&&(Gentamycin)	Pure Culture 3	Pure Culture 6
Gram −ve	*Escherichia coli*	21	09	08
	*Pseudomonas aeruginosa*	20	12	11
Gram +ve	*Staphylococcus aureus*	21	10	12
	*Bacillus subtilis*	19	11	09

**Table 2:** FT-IR spectral analysis of bacterial supernatant-synthesized silver nanoparticles proved the presence of functional groups at a spectral band range of 4000 to 400 cm^−1^.

Spectral Band (cm^−1^)	Functional Group
3450–3320	Hydroxyl group and H-bonded
1635	Amide I (C=O stretching vibration)
1350	N-H bending vibration
1232	C-N stretching vibration

## Data Availability

All the data is incorporated in the manuscript.

## Supplementary Materials

The following supporting information can be downloaded at: https://www.mdpi.com/article/10.3390/antibiotics12010141/s1, Figure S1: (a). Nucleotide sequence of *Lactiplantibacillus plantarum* from buffalo milk using 16s RNA primers. (b). Phylogenetic tree of *Lactiplantibacillus plantarum using* NCBI blast homology search tool.

## References

[1] Goyal R., Macri L.K., Kaplan H.M., Kohn J. (2016). Nanoparticles and nanofibers for topical drug delivery. J. Control. Release.

[2] Camargo P.H.C., Satyanarayana K.G., Wypych F. (2009). Nanocomposites: Synthesis, structure, properties and new application opportunities. Mater. Res..

[3] Wang L., Kafshgari M.H., Meunier M. (2020). Optical Properties and Applications of Plasmonic-Metal Nanoparticles. Adv. Funct. Mater..

[4] Coronado E.A., Encina E.R., Stefani F. (2011). Optical properties of metallic nanoparticles: Manipulating light, heat and forces at the nanoscale. Nanoscale.

[5] Yan Y., Neville A., Dowson D., Williams S., Fisher J. (2009). Effect of metallic nanoparticles on the biotribocorrosion behaviour of Metal-on-Metal hip prostheses. Wear.

[6] Astruc D., Lu F., Aranzaes J.R. (2005). Nanoparticles as Recyclable Catalysts: The Frontier between Homogeneous and Heterogeneous Catalysis. Angew. Chem. Int. Ed..

[7] Myers P.D., Alam T.E., Kamal R., Goswami D.Y., Stefanakos E. (2016). Nitrate salts doped with CuO nanoparticles for thermal energy storage with improved heat transfer. Appl. Energy.

[8] Jain S., Mehata M.S. (2017). Medicinal plant leaf extract and pure flavonoid mediated green synthesis of silver nanoparticles and their enhanced antibacterial property. Sci. Rep..

[9] Balashanmugam P., Kim H.J., Singh V., Kumaran R.S. (2018). Green synthesis of silver nanoparticles using *Ginkgo biloba* and their bactericidal and larvicidal effects. Nanosci. Nanotechnol. Lett..

[10] Balashanmugam P., Jothinathan M.K.D., Murugan R., Palani P., Kalaichelvan P.T., Kim H.J., Singh V., Kumaran R.S. (2017). An in vitro study on the burn wound healing activity of cotton fabrics incorporated with phytosynthesized silver nanoparticles in male Wistar albino rats. Eur. J. Pharm. Sci..

[11] Kumar V., Yadav S.K. (2009). Plant-mediated synthesis of silver and gold nanoparticles and their applications. J. Chem. Technol. Biotechnol..

[12] Thakkar K.N., Mhatre S.S., Parikh R.Y. (2010). Biological synthesis of metallic nanoparticles. Nanomedicine.

[13] Ahmad F., Ashraf N., Ashraf T., Zhou R.B., Yin D.C. (2019). Biological synthesis of metallic nanoparticles (MNPs) by plants and microbes: Their cellular uptake, biocompatibility, and biomedical applications. Appl. Microbiol. Biotechnol..

[14] Noruzi M. (2015). Biosynthesis of gold nanoparticles using plant extracts. Bioprocess Biosyst. Eng..

[15] Loo Y.Y., Rukayadi Y., Nor-Khaizura M.A., Kuan C.H., Chieng B.W., Nishibuchi M., Radu S. (2018). In vitro antimicrobial activity of green synthesized silver nanoparticles against selected gram-negative foodborne pathogens. Front. Microbiol..

[16] Arafa M.G., El-Kased R.F., Elmazar M.M. (2018). Thermoresponsive gels containing gold nanoparticles as smart antibacterial and wound healing agents. Sci. Rep..

[17] Huang H., Qi X., Chen Y., Wu Z. (2019). Thermo-sensitive hydrogels for delivering biotherapeutic molecules: A review. Saudi Pharma. J..

[18] Catauro M., Raucci M.G., De Gaetano F.D., Marotta A. (2004). Antibacterial and bioactive silver-containing Na_2_O × CaO × _2_SiO_2_ glass prepared by solgel method. J. Mater. Sci. Mater. Med..

[19] Crabtree J.H., Burchette R.J., Siddiqi R.A., Huen I.T., Handott L.L., Fishman A. (2003). The efcacy of silver-ion implanted catheters in reducing peritoneal dialysis-related infections. Perit. Dial. Int..

[20] Rani C., Ragunathan C.R., Prasannakumar K. (2010). Biosynthesis of Silver Nano Particles Using L. Acidophilus (Probiotic Bacteria) and its Application. Int. J. Nanotechnol. Appl..

[21] Wilhelm H.H., Ulrich S. (2002). Introduction to pre- and probiotics. Food Res. Int..

[22] Carlos R.S., Luciana P.S.V., Michele R.S., Adriane B.P.M., Caroline T.Y., Juliano D.D.L., Ashok P., Vanete T.S. (2010). The Potential of Probiotics: A Review. Food Technol. Biotechnol..

[23] Cosme-Silva L., Dal-Fabbro R., Cintra L.T.A., Ervolino E., Prado A.S.A.d., Oliveira D.P.d., Marcelos P.G.C.L.d., Gomes-Filho J.E. (2021). Dietary supplementation with multi-strain formula of probiotics modulates inflammatory and immunological markers in apical periodontitis. J. Appl. Oral Sci..

[24] Cosme-Silva L., Dal-Fabbro R., Cintra L.T.A., Ervolino E., Plazza F., Mogami Bomfim S., Duarte P.C.T., Junior V., Gomes-Filho J.E. (2020). Reduced bone resorption and inflammation in apical periodontitis evoked by dietary supplementation with probiotics in rats. Int. Endod. J..

[25] Lehtoranta L., Pitkäranta A., Korpela R. (2014). Probiotics in respiratory virus infections. Eur. J. Clin. Microbiol. Infect. Dis..

[26] Soghra K., Hamideh M.H., Mohammad T., Mohammad R.N., Abbas A.I.F. (2012). Probiotics as an alternative strategy for prevention and treatment of human diseases: A review. Inflamm. Allergy-Drug Targets.

[27] Sahar K., Mohammad R., Hosna H., Mahmoud B., Leila, Mahmoud, K (2017). Isolation and identification of probiotic *Lactobacillus* from local dairy and evaluating their antagonistic effect on pathogens. Int. J. Pharm. Investig..

[28] Losada M.A., Olleros T. (2006). Towards a healthier diet for the colon: The influence of fructo oligosaccharides and *lactobacilli* on intestinal health. Nutr. Res..

[29] Caggianiello G., Kleerebezem M., Spano G. (2016). Exopolysaccharides produced by lactic acid bacteria: From health-promoting benefits to stress tolerance mechanisms. Appl. Microbiol. Biotechnol..

[30] Anjana, Santosh Kumar, T (2022). Bacteriocin-Producing Probiotic Lactic Acid Bacteria in Controlling Dysbiosis of the Gut Microbiota. Front. Cell. Infect. Microbiol..

[31] Rebecca K., Thomas K., James G. (2020). The role of topical probiotics on wound healing: A review of animal and human studies. Int. Wound J..

[32] Ahmadova A., Todorov S.D., Hadji-Sfaxi I., Choiset Y., Rabesona H., Messaoudi S. (2013). Antimicrobial and antifungal activities of *Lactobacillus curvatus* strain isolated from homemade Azerbaijani cheese. Anaerobe.

[33] Babak H., Yousef N., Ali A., Norhafizah A., Dayang R., Rozita R., Abolfazl B., Ahmad Y.K. (2017). Isolation and characterization of probiotics from dairies. Iran. J. Microbiol..

[34] Ben A.K., Vaughan E.E., de Vos W.M. (2007). Advanced molecular tools for the identification of lactic acid bacteria. J. Nutr..

[35] Haghshenas B., Nami Y., Abdullah N., Radiah D., Rosli R., Khosroushahi A.Y. (2014). Anti-proliferative effects of *Enterococcus* strains isolated from fermented dairy products on different cancer cell lines. J. Funct. Foods.

[36] Sikder M., Lead J.R., Chandler G.T., Baalousha M. (2018). A rapid approach for measuring silver nanoparticle concentration and dissolution in seawater by UV-Vis. Sci. Total Environ..

[37] Naseer Q.A., Xuec X., Wang X., Dang S., Din S.U., Kalsoom, Jamil, J (2022). Synthesis of silver nanoparticles using Lactobacillus bulgaricus and assessment of their antibacterial potential. Braz. J. Biol..

[38] Poonam S., Rajani R., Rajendra M. (2021). Dairy Products Mediated Green Synthesis of Silver Nanoparticles: A Comparative Study. JETIR.

[39] Coates J. Interpretation of Infrared Spectra, A Practical Approach. In Encyclopedia of Analytical Chemistry; John Wiley & Sons Ltd, Chichester, UK, 2000; pp. 10815–10837.

[40] Nourbakhsh H., Madadlou A., Emam-Djomeh Z., Wang Y., Gunasekaran S. (2016). One-pot nanoparticulation of potentially bioactive peptides and gallic acid encapsulation. Food Chem..

[41] Vass E., Hollosi M., Besson F., Buchet R. (2003). Vibrational Spectroscopic Detection of Beta- and Gamma-Turns in Synthetic and Natural Peptides and Proteins. Chem. Rev..

[42] Kong J., Yu S. (2007). Fourier Transform Infrared Spectroscopic Analysis of Protein Secondary Structures. Acta Biochim. Biophys. Sin..

[43] Heuer C., Luinge H.J., Lutz E.T., Schukken Y.H., van der Maas J.H., Wilmink H., Noordhuizen J.P. (2001). Determination of Acetone in Cow Milk by Fourier Transform Infrared Spectroscopy for the Detection of Subclinical Ketosis, *J*. Dairy Sci..

[44] Ali B., Khan K.Y., Majeed H., Abid M., Xu L., Wu F., Xu X. (2017). Soymilk-Cow’s milk ACE-inhibiting enzyme modified cheese. Food Chem..

[45] Ashraf S., Abbasi A.Z., Pfeiffer C., Hussain S.Z., Khalid Z.M., Gil P.R., Parak W.J., Hussain I. (2013). Protein-mediated synthesis, pH-induced reversible agglomeration, toxicity and cellular interaction of silver nanoparticles. Colloids Surf. B Biointerfaces.

[46] Kasthuri J., Veerapandian S., Rajendiran N. (2009). Biological synthesis of silver and gold nanoparticles using apiin as reducing agent. Colloids Surf. B.

[47] Wang W., He J., Pan D., Wu Z., Guo Y., Zeng X., Lian L. (2018). Metabolomics analysis of *Lactobacillus plantarum* ATCC 14917 adhesion activity under initial acid and alkali stress. PLoS ONE.

[48] Hidayat M., Abdul R., Rosfarizan M., Uswatun H. (2020). Microbial Mediated Synthesis of Silver nanoparticles by *Lactobacillus plantarum TA4* and Its Antibacterial and, Antioxidant Activity. Appl. Sci..

[49] Hamed B., Soheila H., Pouneh E., Milad A. (2014). Microbial mediated preparation, characterization and optimization of gold nanoparticles. Braz. J. Microbiol..

[50] Saravanan M., Priya A., Raja S., Konathala R. (2017). Biomimetic synthesis of silver nanoparticles from Streptomyces atrovirens and their potential anticancer activity against human breast cancer cells. IET Nanobiotechnol..

[51] Aparajita V., Mohan S.M. (2015). Controllable synthesis of silver nanoparticles using Neem leaves and their antimicrobial activity. J. Radiat. Res. Appl. Sci..

[52] Khalil M.M.H., Ismail E.H., El-Baghdady K.Z., Mohamed D. (2013). Green synthesis of silver nanoparticles using olive leaf extract and its antibacterial activity. Arab. J. Chem..

[53] Yacaman M.J., Ascencio J.A., Liu H.B., Gardea-Torresdey J. (2001). Structure shape and stability of nanometric sized particles. J. Vac. Sci. Technol. B Microelectron. Nanometer Struct. Process. Meas. Phenom..

[54] Li J., Rong K., Zhao H., Li F., Lu Z., Chen R. (2013). Highly selective antibacterial activities of silver nanoparticles against *Bacillus subtilis*. J. Nanosci. Nanotechnol..

[55] Chauhan N., Tyagi A.K., Kumar P., Malik A. (2016). Antibacterial potential of *Jatropha curcas* synthesized silver nanoparticles against food borne pathogens. Front. Microbiol..

[56] Pal S., Tak Y.K., Song J.M. (2007). Does the antibacterial activity of silver nanoparticles depend on the shape of the nanoparticle? A study of the gram-negative bacterium Escherichia coli. Appl. Environ. Microbiol..

[57] Netala V.R., Kotakadi V.S., Nagam V., Bobbu P., Ghosh S.B., Tartte V. (2014). First report of biomimetic synthesis of silver nanoparticles using aqueous callus extract of *Centella asiatica* and their antimicrobial activity. Appl. Nanosci..

[58] Rashid M.M.O., Akhter K.N., Chowdhury J.A., Hossen F., Hussain M.S., Hossain M.T. (2017). Characterization of phytoconstituents and evaluation of antimicrobial activity of silver extract nanoparticles synthesized from *Momordica charantia* fruit extract. BMC Complement. Altern. Med..

[59] Ovais M., Khalil A.T., Raza A., Khan M.A., Ahmad I., Islam N.U., Saravanan M., Ubaid M.F., Ali M., Shinwari Z.K. (2016). Green synthesis of silver nanoparticles via plant extracts: Beginning a new era in cancer theranostics. Nanomedicine.

[60] Singh J., Kaur G., Kaur P., Bajaj R., Rawat M. (2016). A review on green synthesis and characterization of silver nanoparticles and their application: A green nanoworld. World J. Pharm. Pharm. Sci..

[61] Kim S.H., Lee H.S., Ryu D.S., Choi S.J., Lee D.S. (2011). Antibacterial Activity of Silver-nanoparticles Against *Staphylococcus aureus* and *Escherichia coli*. Korean J. Microbiol. Biotechnol..

[62] Hussein R.R.S., Farghali A.A., Hassanein A.H.A., Ibraheem I.B.M. (2017). Biosynthesis of silver nanoparticles by using of the marine alga *Gracilariaparvispora* and its antagonistic efficacy against some common skin infecting pathogens. Aust. J. Basic Appl. Sci..

[63] Reddy N.J., Nagoor Vali D., Rani M. (2014). Evaluation of antioxidant, antibacterial and cytotoxic effects of green synthesized silver nanoparticles by *Piper longum* fruit. Mater. Sci. Eng. C Mater. Biol. Appl..

[64] Nayak D., Pradhan S., Ashe S., Nayak B. (2015). Biologically synthesized silver nanoparticles from three diverse family of plant extracts and their anticancer activity against epidermoid A431 carcinoma. J. Colloid Interface Sci..

[65] Amro N.A., Kotra L.P., Wadu-Mesthrige K., Bulychev A., Mobashery S., Liu G. (2000). High-resolution atomic force microscopy studies of the *Escherichia coli* outer membrane: Structural basis for permeability. Langmuir.

[66] Mirzajani F., Ghassempour A., Aliahmadi A., Esmaeili M.A. (2011). Antibacterial effect of silver nanoparticles on *Staphylococcus aureus*. Res. Microbiol..

[67] Yamanaka M., Hara K., Kudo J. (2005). Bactericidal actions of a silver ion solution on *Escherichia coli*, studied by energy-filtering transmission electron microscopy and proteomic analysis. Appl. Environ. Microbiol..

[68] Lok C.N., Ho C.M., Chen R., He Q.Y., Yu W.Y., Sun H. (2006). Proteomic analysis of the mode of antibacterial action of silver nanoparticles. J. Proteome. Res..

[69] Carlson C., Hussain S.M., Schrand A.M., Braydich-Stolle L.K., Hess K.L., Jones R.L., Schlager J.J. (2008). Unique cellular interaction of solver nanoparticles: Size-dependent generation of reactive oxygen species. J. Phys. Chem. B.

[70] Liu Y., Wu C., Wang H. (2010). Impact of silver nanoparticles on human cells: Effect of particle size, shape and physical properties. Nanotoxicology.

[71] Xia X., Zeng J., Moran C.H., Xia Y. (2012). Recent developments in shape-controlled synthesis of silver nanocrystals. J. Phys. Chem. C.

[72] Hosseinidoust Z., Basenet M., van de Ven T.G. (2016). Tufenkji, N. One-pot green synthesis of anisotropic silver nanoparticles. Environ. Sci. Nano.

[73] Helmlinger J., Sengstock C., Groß-Heitfeld C., Mayer C., Schildhaue T.A., Koller M., Epple M. (2016). Silver nanoparticles with different size and shape: Equal cytotoxicity, but different antibacterial effects. RSC Adv..

[74] Akter M., Sikder M.T., Rahman M., Ullah A.K., Hossain K.F., Banik S., Hosokawa T., Saito T., Kurasaki M. (2018). A systematic review on silver nanoparticles-induced cytotoxicity: Physicochemical properties and perspectives. J. Adv. Res..

[75] Jaiswal S., Mishra P. (2018). Antimicrobial and antibiofilm activity of curcumin-silver nanoparticles with improved stability and selective toxicity to bacteria over mammalian cells. Med. Microbiol. Immunol..

[76] Baygar T. (2019). Characterization of silk sutures coated with propolis and biogenic silver nanoparticles (AgNPs); an eco-friendly solution with wound healing potential against surgical site infections (SSIs). Turk. J. Med. Sci..

[77] Daniela M.S., Claudia Y.H., Adnan Y.T., Maricê N.d.O. (2011). Evaluation of different selective media for enumeration of probiotic micro-organisms in combination with yogurt starter cultures in fermented milk. Afr. J. Microbiol. Res..

[78] Gayathri V., Nivedha S., Pujita V., Ivo R.S. (2020). Green synthesis of copper nanoparticles using bracts of *Musa paradisiaca* (Monthan) and study of its antimicrobial and antioxidant activity. Res. J. Pharm. Technol..

[79] Arumugam T., Senthil Kumar P., Hemavathy R.V., Swetha V., Karishma Sri R. (2018). Isolation, structure elucidation and anticancer activity from *Brevibacillus brevis* EGS 9 that combats Multi Drug Resistant actinobacteria. Microb. Pathogen..

[80] Balashanmugam P., Prabhu D., Devasena T., Hak J.S., Kim K.W., Jung Y.S., Song H.J., Kim H.J., Kumaran R.S. (2020). Facile synthesis of silver nanoparticles using Asian spider flower and its in vitro cytotoxic activity against human breast carcinoma cells. Processes.

[81] Vijayakumar G., Kesavan H., Kannan A., Arulanandam D., Kim J.H., Kim K.J., Song H.J., Kim H.J., Rangarajulu S.K. (2021). Phytosynthesis of Copper Nanoparticles Using Extracts of Spices and Their Antibacterial Properties. Processes.

[82] Kouhkan M., Ahangar P., Babaganjeh L., Ashrafi M. (2020). Biosynthesis of Copper Oxide nanoparticles Using *Lactobacillus casei* Subsp. *Casei* and its Anticancer and Antibacterial Activities. Curr. Nanosci..

[83] Alam H., Nafeesa K., Mohammad A., Syed A., Saravanan M. (2019). Synthesis of Selenium nanoparticles Using Probiotic Bacteria *Lactobacillus acidophilus* and Their Enhanced Antimicrobial Activity Against Resistant Bacteria. J. Clust. Sci..

[84] Amr T.M.S., Ahmad S.A., Hessa A.B., Khalid A.A.R. (2014). Production of silver nanoparticles with strong and stable antimicrobial activity against highly pathogenic and multidrug resistant bacteria. Sci. World J..

[85] Suriati G., Nureen N. (2016). Morphology and optical properties of AgNPs: Effect of reducing agent to surfactant ratio. ARPN J. Eng. Appl. Sci..

[86] Pannerselvam B., Thiyagarajan D., Pazhani A., Thangavelu K.P., Kim H.J., Rangarajulu S.K. (2021). Copperpod Plant Synthesized AgNPs Enhance Cytotoxic and Apoptotic Effect in Cancer Cell Lines. Processes.

[87] Mouritzen M.V., Andrea A., Qvist K., Poulsen S.S., Jenssen H. (2019). Immunomodulatory potential of Nisin A with application in wound healing. Wound Repair Regen..

[88] Ong J.S., Taylor T.D., Yong C.C., Khoo B.Y., Liong M.T. (2019). *Lactobacillus plantarum* USM8613 Aids in Wound Healing and Suppresses Staphylococcus aureus Infection at Wound Sites. Probiotics Antimicrob. Proteins.

[89] Nicole C.A., Magda B.S., Abdu F.A. (2008). Growth and Maintenance of Vero Cell Lines. Curr. Protoc. Microbiol..

[90] Mossman T. (1983). Rapid Colorimetric Assay for Cellular Growth and Survival: Application to Proliferation and Cytotoxicity Assays. J. Immunol. Methods.

[91] Malarvizhi G., Karpagam S.C. (2017). Evaluating the natural fibre reinforced polymer biocomposite for the development of novel wound dressing materials. Int. J. Res. Ayurveda. Pharm..

[92] Felice F., Zambito Y., Belardinelli E. (2015). Effect of different chitosan derivatives on in vitro scratch wound assay: A comparative study. Int. J. Biol. Macromol..

