# Supplementary material for: In Vitro Antibacterial and Wound Healing Activities Evoked by Silver Nanoparticles Synthesized through Probiotic Bacteria
Source: Antibiotics (Basel). 2023 Jan 10;12(1):141. doi: 10.3390/antibiotics12010141 (PMC9854575; doi:10.3390/antibiotics12010141)
Supplement: Supplementary file 1 [file antibiotics-12-00141-s001.zip › antibiotics-2120190-supplementary.pdf]

CTCTTCTGCCTCAAGTTTCCCAGTTTCCGATGCACTTCTTCGGTTGAGCCGAAGGCTTT
CACATCAGACTTAAAAAACCGCCTGCGCTCGCTTTACGCCCAATAAATCCGGACAACGC
TTGCCACCTACGTATTACCGCGGCTGCTGGCACGTAGTTAGCCGTGGCTTTCTGGTTAA
ATACCGTCAATACCTGAACAGTTACTCTCAATATGTTCTTCTTTAACAACAGAGTTTTA
CGAGCCGAAACCCTTCTTCACTCACGCGGCGTTGCTCCATCAGACTTTCGTCCATTGTG
GAAGATTCCCTACTGCTGCCTCCCGTAGGAGTTTGGGCCGTGTCTCAGTCCCAATGTGG
CCGATTACCCTCTCAGGTCGGCTACGTATCATTGCCATGGTGAGCCGTTACCTACCATC
TAGCTAATACGCCGCGGGACCATCCAAAAGTGATAGCCGAAGCCATCTTTCAAACTCGG
ACCATGCGGTCCAAGTTGTTATGCGGTATTAGCATCTGTTTCCAGGTGTTATCCCCCGC
TTCGGGCAGGTTTCCCACGTGTTACTCACCAGTTCGCCACTCACCAAATGTAAATCATG
ATGCAAGCACCAATCAATACCAGAGTTCGTTCGACTTGCATGTATTAGGCCGCC

(**a**)

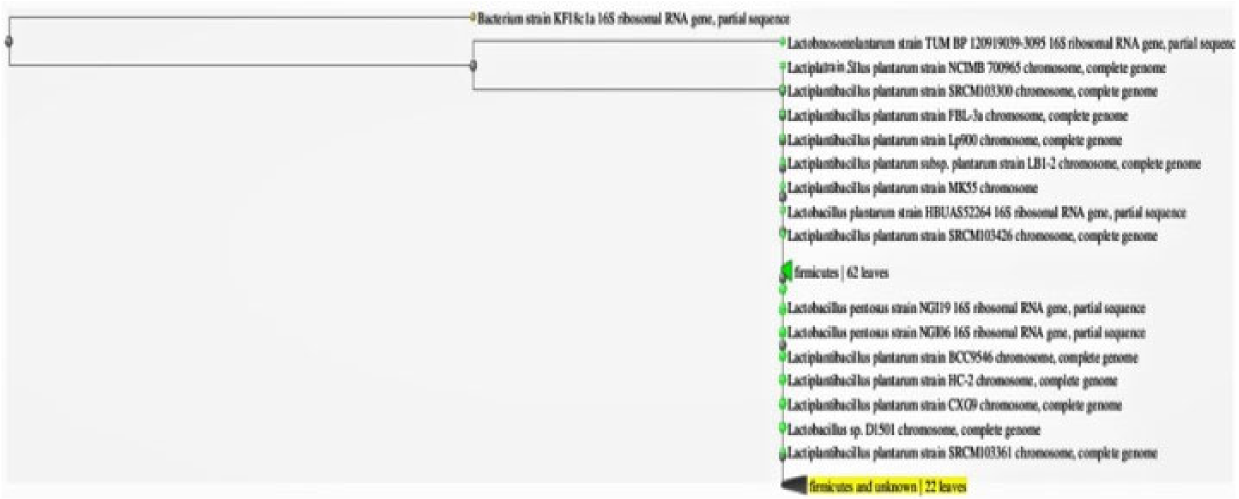

(**b**)

Figure S1. (**a**) Nucleotide sequence of *Lactiplantibacillus plantarum* from buffalo milk using 16s RNA primers. (**b**) Phylogenetic tree of *Lactiplantibacillus plantarum using* NCBI blast homology search tool.
